# Synteny plot quality control with SyntenyQC

**DOI:** 10.1093/bioinformatics/btaf626

**Published:** 2025-11-13

**Authors:** Timothy D J Kirkwood, Jack A Connolly, Ee Lui Ang, Huimin Zhao, Eriko Takano, Rainer Breitling

**Affiliations:** Manchester Institute of Biotechnology, Department of Chemistry, School of Natural Sciences, Faculty of Science and Engineering, University of Manchester, Manchester M1 7DN, United Kingdom; Manchester Institute of Biotechnology, Department of Chemistry, School of Natural Sciences, Faculty of Science and Engineering, University of Manchester, Manchester M1 7DN, United Kingdom; Synthetic Biology Translational Research Program, Yong Loo Lin School of Medicine, National University of Singapore, Singapore 117597, Singapore; Singapore Institute of Food and Biotechnology Innovation (SIFBI), Agency for Science, Technology and Research (A*STAR), Singapore 138669, Singapore; Department of Chemical and Biomolecular Engineering, University of Illinois at Urbana-Champaign, Urbana, IL 61801, United States; Carl R. Woese Institute for Genomic Biology, University of Illinois at Urbana-Champaign, Urbana, IL 61801, United States; NSF Molecular Maker Lab Institute, University of Illinois at Urbana-Champaign, Urbana, IL 61801, United States; NSF iBiofoundry, University of Illinois at Urbana-Champaign, Urbana, IL 61801, United States; Manchester Institute of Biotechnology, Department of Chemistry, School of Natural Sciences, Faculty of Science and Engineering, University of Manchester, Manchester M1 7DN, United Kingdom; Singapore Institute of Food and Biotechnology Innovation (SIFBI), Agency for Science, Technology and Research (A*STAR), Singapore 138669, Singapore; Singapore Integrative Biosystems and Engineering Research (SIBER) Strategic Research Translational Thrust (SRTT), Agency for Science, Technology and Research (A*STAR), Singapore 138635, Singapore; Manchester Institute of Biotechnology, Department of Chemistry, School of Natural Sciences, Faculty of Science and Engineering, University of Manchester, Manchester M1 7DN, United Kingdom; Bioinformatics Institute (BII), Agency for Science, Technology and Research (A*STAR), Singapore 138671, Singapore

## Abstract

**Summary:**

SyntenyQC is a data pre-processing tool for the construction of synteny plots. It supports genomic data collection, annotation and dereplication to facilitate (and in some cases fundamentally enable) the construction of informative synteny plots.

**Availability and implementation:**

SyntenyQC is a command line app developed using Python version 3.10 and tested using pytest. SyntenyQC is available on PyPI (https://pypi.org/project/SyntenyQC) under the MIT License, along with a detailed user tutorial. Package tests can be viewed at https://github.com/Tim-Kirkwood/SyntenyQC.

## 1 Introduction

The synteny plot is a commonly used comparative genomics tool (Gilchrist and Chooi 2021). Synteny plots consist of multiple tracks, each of which represents a separate genome or genomic subsequence. Synteny plots can be drawn at either genome or neighbourhood scales, with the latter being the focus of this work. Each track of a neighbourhood-scale synteny plot represents genes as arrows, whose length corresponds to gene length and direction to gene strand. Typically, links are drawn across tracks between genes that have a shared relationship (e.g. homology), although richer information such as GC content can be included (Hackl *et al.* 2024). Such neighbourhood analyses can (i) indicate mis-annotated gene sequences, (ii) indicate co-selective relationships between specific neighbourhood genes, and (iii) suggest whether a given neighbourhood appears to have recently arrived via horizontal gene transfer (Amos *et al.* 2017). These questions are particularly pertinent in the context of biosynthetic gene clusters (BGCs), which are clustered genes that encode pathways responsible for the production of clinically and economically relevant natural products (Jensen 2016). Indeed, syntenic analysis and/or plots are central to several BGC databases and annotation tools (Navarro-Muñoz *et al.* 2020, Blin *et al.* 2024, 2025, Salamzade *et al.* 2025, Zdouc *et al.* 2025, Zhang *et al.* 2025).

Various ‘low-code’ tools have been developed for synteny plot construction. These typically utilize a command line (Sullivan *et al.* 2011, Gilchrist and Chooi 2021, Gilchrist *et al.* 2021) and/or graphical (Sullivan *et al.* 2011, Medema *et al.* 2013, van den Belt *et al.* 2023) user interface to open up flexible, user-driven analyses without requiring programming proficiency. However, challenges remain for users of these tools. Firstly, from a logistical perspective, it is often useful to annotate synteny plot tracks according to the neighbourhoods that they represent, e.g. via an NCBI accession or organism name. However, this can be challenging to automate as current tools do not retain taxonomically relevant information for identified neighbourhoods (Gilchrist and Chooi 2021, van den Belt *et al.* 2023).

**Figure 1.**
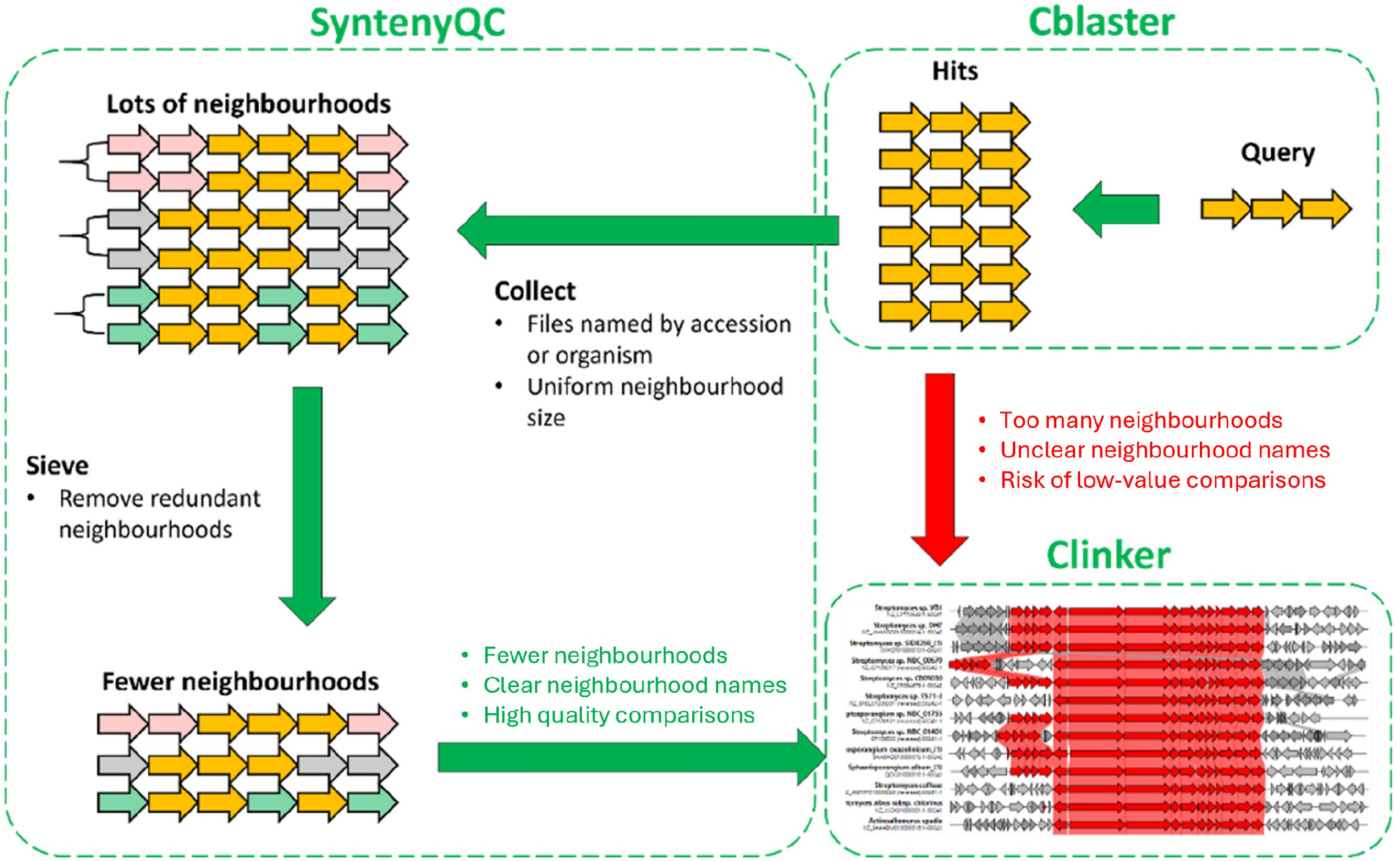
Integrating SyntenyQC into a synteny plot creation workflow. First, cblaster is used to find records (i.e. genomes/contigs) containing a user-specified number of clustered hits to a user-supplied query. The loci of these homologs in each hit record are used to define a neighbourhood for each record, which is extracted using the SyntenyQC Collect subcommand. Files are named according to accession or organism name, facilitating automated annotation of the final synteny plot. Similar neighbourhoods (black braces) are filtered to remove redundant neighbourhoods, with what constitutes ‘similar’ specified by the user in the form of a similarity threshold. Finally, the collected, sieved neighbourhoods are fed into clinker to create the final synteny plot.

More importantly, identifying neighbourhoods for synteny plot analysis can be complicated by redundancy at the neighbourhood (e.g. similar strains) and database (e.g. multiple copies of the same genome) levels. In practice, this means that many neighbourhoods will be highly similar, and thus uninformative in the context of a synteny plot (which typically requires a degree of neighbourhood diversity for the comparison of conserved and non-conserved neighbourhood elements). The inclusion of redundant neighbourhoods also increases computational workloads and may preclude the creation of synteny plots altogether—e.g. CAGECAT (van den Belt *et al.* 2023) imposes a limit of 50 neighbourhoods per synteny plot visualization. There is thus a broken link between stand-alone tools that can source neighbourhoods for synteny plot creation, and the creation/interpretation of synteny plots themselves (Fig. 1).

These challenges are addressed by some BGC annotation tools via genome-level (Blin *et al.* 2024, 2025) or neighbourhood-level (Cediel-Becerra *et al.* 2025, Salamzade *et al.* 2025) redundancy filters. However, limited quality control options exist for low-code synteny plot construction. An exception, CAGEcleaner (De Vrieze *et al.* 2025), is uniquely well placed for BGC-specific, host-centric analyses (e.g. pan-genomic analysis of hosts that contain a given BGC). However, CAGEcleaner’s genome-level redundancy filter is not optimal for synteny plot creation—genomic variation does not guarantee neighbourhood variation, and vice versa, particularly in scenarios of pervasive horizontal gene transfer, which are often of particular interest for this kind of analysis (see Results Section 1, available as supplementary data at *Bioinformatics* online and Fig. 5, available as supplementary data at *Bioinformatics* online). Additionally, CAGEcleaner is not a cross-platform tool, is strictly coupled to cblaster (Gilchrist *et al.* 2021), and applies a complex workflow with multiple parameters to optimize. CAGEcleaner’s similarity metric (Average Nucleotide Identity; ANI) fails at similarities below 82% (De Vrieze *et al.* 2025)—whilst acceptable for genome-scale comparisons, this could hinder comparisons of the smaller contigs commonly observed in most sequence databases. Indeed, CAGEcleaner performs poorly on low-quality assemblies (De Vrieze *et al.* 2025), which is not ideal as neither cblaster nor CAGEcleaner perform sequence quality control.

## 2 Features

SyntenyQC is a Python app for the quality control and automated curation of neighbourhoods prior to synteny plot creation. It offers two subcommands, Collect and Sieve. Sieve is used to remove redundant neighbourhoods from a user-specified folder of GenBank-format neighbourhood files and is thus not tied to any specific upstream tool. However, in recognition of the increasing popularity of cblaster (Gilchrist *et al.* 2021), we also provide an explicit integration point for the cblaster output via Collect, which downloads (optionally extended) cblaster-identified genomic neighbourhoods from NCBI. These neighbourhoods are written to local GenBank-format files, which are named according to either NCBI Accession or Organism.

## 3 Usage

As an example use case, we applied the workflow shown in Fig. 1 to the classic actinorhodin BGC, using entry BGC0000196 in the ‘Minimum Information about a Biosynthetic Gene cluster’ (MIBiG) database of verified clusters as a reference (Zdouc *et al.* 2025). The use of cblaster (Gilchrist and Chooi 2021) highlighted 173 neighbourhoods containing potential cluster homologs—unfortunately, this number of neighbourhoods was too high for synteny plot creation using clinker (Gilchrist and Chooi 2021). Processing with SyntenyQC Collect and Sieve reduced the number of neighbourhoods to 139 and 22, respectively. The 22 neighbourhoods identified with Sieve were used to create a synteny plot with clinker, shown in Fig, 2. See Methods Section 1, available as supplementary data at *Bioinformatics* online, for further information on cblaster and SyntenyQC processing, and Fig. 3 for a comparison of neighbourhood numbers in the context of other *Streptomyces coelicolor* BGCs.

**Figure 2.**
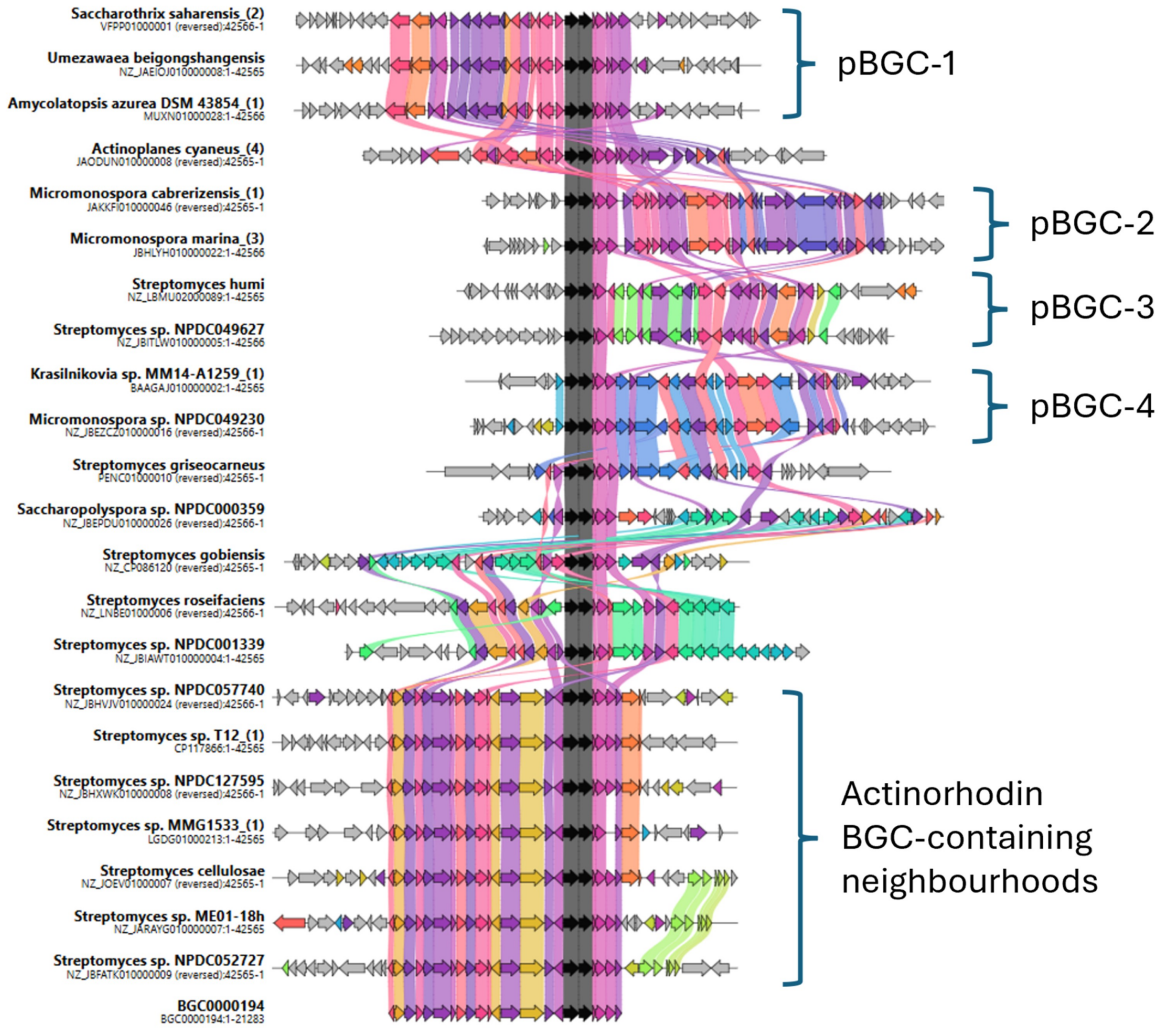
A synteny plot for the actinorhodin BGC. Each track represents a separate genomic neighbourhood. The bottom track shows the genes within actinorhodin MIBiG entry BGC000194, which is used as the BGC reference in this plot. Coloured links indicate genes encoding proteins which fall within the same homology group. Black links indicate genes that encode proteins within the same homology group as SCO5087 and SCO5088, the only genes annotated as core biosynthetic within MIBiG entry BGC0000194. These genes encode the actinorhodin polyketide beta-ketoacyl synthase alpha and beta subunits, respectively. The annotation ‘pBGC’ (putative BGC) highlights neighbourhoods in different strains and/or genera, which share a clear set of genes with conserved homology and synteny.

**Figure 3.**
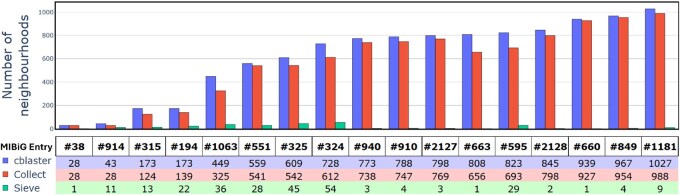
SyntenyQC curation dramatically reduces the number of neighbourhoods associated with a cblaster homology search. ‘MIBiG Entry’ refers to *Streptomyces coelicolor* entries in the MIBiG database of experimentally characterized biosynthetic gene clusters (BGCs). The ‘cblaster’ values refer to the number of hit records identified by a cblaster search for homologs to a given BGC. ‘Collect’ refers to the number of neighbourhoods remaining after the SyntenyQC Collect subcommand is called on the cblaster records. ‘Sieve’ refers to the number of neighbourhoods that remain after the SyntenyQC Sieve subcommand is called on the collected neighbourhoods.

Automated annotation of the synteny plot tracks allows us to see that the actinorhodin BGC can only be observed within the *Streptomyces* genus (Fig. 2). However, several potentially related putative BGCs (pBGCs) can be observed outside of the *Streptomyces* genus. These pBGCs are conserved across multiple species and, in the case of pBGC-1 and pBGC-4, genera. Due to the cblaster search parameters (see Methods Section 1, available as supplementary data at *Bioinformatics* online), each pBGC also contained genes that fell within the same homology groups as the (core biosynthetic) actinorhodin polyketide beta-ketoacyl synthase alpha and beta subunits. This supports the hypothesis that these pBGCs are related to the actinorhodin BGC.

To illustrate the effectiveness with which SyntenyQC can remove redundant neighbourhoods, cluster homologs were identified and dereplicated for a test dataset of *Streptomyces coelicolor* BGCs extracted from the MIBiG database of validated BGCs (Zdouc *et al.* 2025) (see Methods Section 1, available as supplementary data at *Bioinformatics* online). The binary file from each cblaster run was then processed using the SyntenyQC Collect sub-command, with a neighbourhood length of twice the BGC length. This resulted in the loss of multiple cblaster neighbourhoods that were small or dispersed beyond the specified neighbourhood length (Fig. 3 and Fig. 1B, available as supplementary data at *Bioinformatics* online). The folder of collected GenBank files was then processed with SyntenyQC Sieve, with a minimum similarity filter of 0.7 and the search strategy outlined in Methods Section 2, available as supplementary data at *Bioinformatics* online. This removed an average of 95% (nearest whole figure) of the neighbourhoods identified by cblaster (standard deviation 7%, nearest whole figure). The raw neighbourhood counts from cblaster, Collect and Sieve are shown in Fig. 3. See Table 1, available as supplementary data at *Bioinformatics* online and Fig. 7, available as supplementary data at *Bioinformatics* online for more information on SyntenyQC performance metrics and an exemplary analysis of Sieve information loss.

Finally, in Results Section 1, available as supplementary data at *Bioinformatics* online, we show that under default settings in the published case studies (De Vrieze *et al.* 2025), CAGEcleaner (i) removes diverse neighbourhoods, despite its hit recovery approaches and (ii) fails to remove many redundant neighbourhoods. We also highlight how high internal homology can hinder Sieve filtering in Results Section 2, available as supplementary data at *Bioinformatics* online.

## 4 Methods

### 4.1 Collect

The ‘Collect’ subcommand represents a direct integration point for cblaster (Gilchrist *et al.* 2021), which has become widely used to find putative BGC homologs and derivatives. ‘Collect’ is used to parse the cblaster results binary file, download neighbourhoods from NCBI (each of a user-defined span and centred on the corresponding cblaster-defined neighbourhood), and then write these neighbourhoods to a local file in GenBank format (Fig. 1A, available as supplementary data at *Bioinformatics* online). Files are named according to either organism or accession (as specified by the user), with the expectation that these filenames will be used to name the associated tracks by whichever synteny visualization software is used, as is done by clinker (Gilchrist and Chooi 2021). This subcommand thus facilitates the extraction of defined neighbourhoods from a cblaster results file and the exclusion of (i) neighbourhoods that are too small (e.g. fragmented contigs) and (ii) neighbourhoods that are too large (indicating cblaster has found hits that are not clustered within the defined neighbourhood span)—see Fig. 1B, available as supplementary data at *Bioinformatics* online.

### 4.2 Sieve

The ‘Sieve’ subcommand applies a graph-based algorithm to remove redundant neighbourhoods, in recognition of the fact that neighbourhood similarity is a non-transitive property—two neighbourhoods may be highly similar to each other, yet differ in terms of their relative similarity to a third neighbourhood (Fig. 2, available as supplementary data at *Bioinformatics* online).

First, all neighbourhoods undergo an all-vs-all BLASTP using DIAMOND (Buchfink *et al.* 2021) to identify reciprocal best hits between every pair of neighbourhoods. This is used to generate a similarity score for every pair of neighbourhoods, representing the proportion of genes in the smallest neighbourhood that have a reciprocal best hit within the larger neighbourhood (Fig. 3, available as supplementary data at *Bioinformatics* online). These scores then define edges in a graph with the neighbourhoods as nodes, which is pruned according to Algorithm 1, available as supplementary data at *Bioinformatics* online to remove neighbourhoods that exceed a user defined similarity (default 0.7) to another neighbourhood within the graph (Fig. 3, available as supplementary data at *Bioinformatics* online). Once the graph has been pruned, the surviving neighbourhoods are written to a results folder in GenBank format. Similarity filters above 0.7 typically generate more redundant plots, but large neighbourhoods extending far beyond core conserved regions (such as a BGC) may need lower similarity filters.

To help with optimization of the graph pruning parameters and illustrate a given neighbourhood space, the graph is also written to the results folder as an interactive html file (Fig. 4, available as supplementary data at *Bioinformatics* online), as is a histogram of all non-zero edge values present within the graph. The graph allows users to compare neighbourhood redundancy to host similarity by looking at the diversity of hosts within a given sub-network of the graph. The histogram indicates how changing the similarity filter might impact graph topology—if a similarity filter is 0.7, and the histogram shows most inter-neighbourhood similarities are close to 1, then increasing the similarity filter would not be expected to change the graph structure in a meaningful way (and using Collect to gather larger neighbourhoods that extend beyond conserved regions might be a better approach).

## 5 Conclusion

In this work, we introduced SyntenyQC, a tool for the pre-processing of genomic neighbourhoods prior to the construction of neighbourhood-scale synteny plots. We then showed how SyntenyQC can be used to collect and objectively reduce the number of neighbourhoods used to construct a given synteny plot, using a collection of verified biosynthetic gene clusters as a test dataset.

## Supplementary Material

btaf626_Supplementary_Data

## Supplementary data

Supplementary Information is available at *Bioinformatics* online. Supplementary Data is available at https://github.com/Tim-Kirkwood/SyntenyQC_application_note
