# Supplementary material for: Synteny plot quality control with SyntenyQC
Source: Bioinformatics. 2025 Nov 13;41(12):btaf626. doi: 10.1093/bioinformatics/btaf626 (PMC12721865; doi:10.1093/bioinformatics/btaf626)
Supplement: btaf626_Supplementary_Data [file btaf626_supplementary_data.pdf]

Synteny Plot quality control with SyntenyQC

Authors: Timothy D. J. Kirkwood[1], Jack A. Connolly[1], Ee Lui Ang[2,3], Huimin Zhao[6,7,8,9], Eriko Takano[1,3,4] and Rainer Breitling[1,5]

[1] Manchester Institute of Biotechnology, Department of Chemistry, School of Natural Sciences, Faculty of Science and Engineering, University of Manchester, 131 Princess Street, Manchester, M1 7DN, UK

[2] Synthetic Biology Translational Research Program, Yong Loo Lin School of Medicine, National University of Singapore, 10 Medical Drive, 117597, Singapore ,

[3] Singapore Institute of Food and Biotechnology Innovation (SIFBI), Agency for Science, Technology and Research (A*STAR), 31 Biopolis Way, Nanos #04-01, 138669, Singapore

[4] Singapore Integrative Biosystems and Engineering Research (SIBER) Strategic Research Translational Thrust (SRTT), Agency for Science, Technology and Research (A*STAR), 2 Fusionopolis Way, Kinesis, #8-05, 138635, Singapore

[5] Bioinformatics Institute (BII), Agency for Science, Technology and Research (A*STAR), 30 Biopolis Street, #07-01 Matrix, 138671, Singapore

[6] Department of Chemical and Biomolecular Engineering, University of Illinois at Urbana-Champaign, 600 South Mathews Avenue, Urbana, 61801, Illinois USA

[7] Carl R. Woese Institute for Genomic Biology, University of Illinois at Urbana-Champaign, 1206 W. Gregory Dr., 61801, Illinois USA

[8] NSF Molecular Maker Lab Institute, University of Illinois at Urbana-Champaign, 1206 W. Gregory Dr., 61801, Illinois USA

[9] NSF iBiofoundry, University of Illinois at Urbana-Champaign, 1206 W. Gregory Dr., 61801, Illinois USA

*Corresponding authors. timothy.kirkwood@manchester.ac.uk, Rainer_Breitling@bii.a-star.edu.sg

Contents

Synteny Plot quality control with SyntenyQC 1

Contents 1

Supplementary Results 2

S1: Benchmarking SyntenyQC against CAGEcleaner 2

S2: High internal homology can hinder Sieve filtering 3

Supplementary Methods 4

S1: Collecting a BGC test data set and processing with SyntenyQC 4

S2: SyntenyQC Sieve search strategy 5

S3: Comparing SyntenyQC and CAGEcleaner 5

Supplementary Figures 6

Supplementary Figure 1: The Collect subcommand. 6

Supplementary Figure 2: Region similarity is non-transitive. 7

Supplementary Figure 3: The Sieve subcommand. 7

Supplementary Figure 4: The neighbourhood graph for actinorhodin MIBIG entry BGC000194. 8

Supplementary Figure 5: Genomic variation does not guarantee neighbourhood variation. 9

Supplementary Figure 6: SyntenyQC Sieve filtering can be less effective on neighbourhoods with high levels of internal redundancy. 10

Supplementary Figure 7: Information loss associated with the Sieve Algorithm. 11

Supplementary Tables 12

Supplementary Table 1: Performance metrics for SyntenyQC. 12

Supplementary Algorithms 23

Algorithm 1 23

Supplementary Bibliography 23

Supplementary Results

S1: Benchmarking SyntenyQC against CAGEcleaner

When comparing SyntenyQC to CAGEcleaner (De Vrieze et al., 2025), we focussed on two characteristics – disk space usage (as reported in De Vrieze et al. (2025)) and neighbourhood filtering behaviour. Run times are provided as a guide for the user (**Supplementary Table 1**), but are not suitable for direct comparison with those reported by De Vrieze et al. (2025) - our tests were performed on a Windows 13th Gen Intel(R) Core(TM) i5-1335U laptop with half the cores (10) and RAM (16GB) of the system used by De Vrieze et al. 2025, so we expect runtimes to be considerably slower on our system. We considered replicating the work of De Vrieze et al. 2025 on our device, but CAGEcleaner is not compatible with the Windows operating system.

To perform benchmarking, we took the CAGEcleaner-filtered and unfiltered cblaster binary files for case studies 1 (Actinorhodin) and 2 (*Staphylococcus*) as described by De Vrieze et al. 2025. Each file was processed with Collect and then Sieve – see **Supplementary Methods** section S3 and **Supplementary Table 1**. Run times were taken from the associated SyntenyQC logs, and size on disk was manually determined for the output of each command using TreeSize.

In general, SyntenyQC requires less disk space than CAGEcleaner (**Supplementary Table 1**), although the difference is dependent on how many commands are used to make up the tiered search strategy we recommend (see **Supplementary Methods** section S2). All SyntenyQC Sieve and Collect commands used to process the unfiltered cblaster binary file for Case Study 1 (Actinorhodin) generated files requiring a total of 5.97GB disk space, which is considerably less than the 28.5 GB required by CAGEcleaner (De Vrieze et al., 2025). Similarly, the SyntenyQC files generated for Case Study 2 (*Staphylococcus*) required 0.782 GB of disk space, which is less than the 1.2GB required by CAGEcleaner (De Vrieze et al., 2025).

In terms of filtering behaviour, CAGEcleaner fails to remove a large proportion of redundant neighbourhoods with default settings (if one accepts “over 70% of

bidirectional best BLASTP hits" as a definition of redundancy). In Case Study 1 (Actinorhodin), CAGEcleaner reduced the 8934 cblaster hits down to 4847 (46% reduction). SyntenyQC Sieve reduced the 8934 hits to 1434 (84% reduction). When SyntenyQC Sieve was applied to the CAGEcleaner filtered neighbourhoods, only 1390 neighbourhoods remained. This indicates that 44 neighbourhoods were considered non-redundant at the neighbourhood level by SyntenyQC Sieve, but were lost by CAGEcleaner filtering at the genome level. This is undesirable for typical applications such as BGC boundary inference, as the 44 lost neighbourhoods were diverse and thus informative.

In Case Study 2 (*Staphylococcus*), which started with 1146 hits, we similarly see a reduction in the number of neighbourhoods remaining following SyntenyQC Sieve (9) relative to CAGEcleaner (22) processing. Moreover, the neighbourhoods retained by CAGEcleaner are highly redundant (**Supplementary Figure 5**) - when SyntenyQC Sieve was applied to the 22 CAGEcleaner filtered neighbourhoods, only 3 neighbourhoods remained. On the other hand, this indicates that 6 neighbourhoods were considered non-redundant at the neighbourhood level by SyntenyQC Sieve but filtered out by CAGEcleaner due to genome-level variation – this is a much larger proportion of information loss (66% of the 9 diverse neighbourhoods identified by SyntenyQC) than was seen in Case Study 1.

S2: High internal homology can hinder Sieve filtering

Supplementary Figure 6 shows the 9-neighbourhood synteny plot created following SyntenyQC Sieve-filtering of the 1146 42.5kb neighbourhoods from Case Study 2 (*Staphylococcus*), without CAGEcleaner processing. This figure serves as an illustrative example for scenarios where Sieve filtering may be overly lax. If we compare "*Staphylococcus sp. HMSC55D02*" (D02) and "*Staphylococcus sp. HMSC077E12*" (E12), we can see that E12 is almost a complete subset of D02 (and others) in terms of homolog composition and so should have been removed by SyntenyQC Sieve. However, upon further inspection, we observed that many of the upstream proteins in E12 could not be established as reciprocal best matches of a protein in D02 (**Supplementary Figure 6**, bottom). This was particularly apparent in E12 proteins that had (i) relatively strong alignments to other proteins within E12, and (ii) multiple hits in D02, suggesting a

likely scenario where a forward hit to a D02 protein was supplanted in the reverse search by an E12 homolog of the initial query. This is compounded by the presence of proteins without sequence information due to annotation inaccuracies – these proteins are not considered by Sieve in its analysis, but are inferred by clinker when building synteny plots (Gilchrist & Chooi, 2021). Thus, if the actual best hit of a given protein is misannotated and has no sequence information, a best hit will not be established, and such misannotated proteins will not be included when calculating neighbourhood sizes for the Sieve similarity score. While such a scenario of internal gene duplication is rare, it illustrates a general limitation of synteny analysis and should be kept in mind when interpreting specific results.

Supplementary Methods

S1: Collecting a BGC test data set and processing with SyntenyQC

The MIBIG database (Zdouc et al., 2025) of verified Biosynthetic Gene Clusters (BGCs) was searched for entries from *Streptomyces coelicolor*. Each BGC protein set was used as a query in a cblaster (Gilchrist et al., 2021) search to identify putative BGC homologs. All hit neighbourhoods were required to have N unique hits to the BGC query, where N was either 5 or half the number of proteins in the query (whichever was larger), and all core biosynthetic proteins were required. For queries with 5 or fewer proteins, N was the number of proteins encoded by the BGC, and no specific proteins were required. Hits were restricted to those within Actinomycete genomes.

The commands used for cblaster, SyntenyQC Collect, and SyntenyQC Sieve are given in **Supplementary Data 1**. The fasta-format query files used for cblaster are given in **Supplementary Data 2**. The binary files output by cblaster are available at **Supplementary Data 3**. The GenBank files generated by SyntenyQC Collect and Sieve are available upon request. All supplementary files are available at Tim-Kirkwood/SyntenyQC_application_note.

S2: SyntenyQC Sieve search strategy

A recursive Sieve search strategy was employed to reduce disk space requirements and run time, with the output of a less sensitive Sieve filter being used as the input of a subsequent more sensitive Sieve filter (**Supplementary Data 1**, **Supplementary Data 4**, **Supplementary Table 1**).

This involved using DIAMOND with a "fast" alignment mode, which typically misses alignments with less than 90% identity, and setting a low max_target_seqs value. This will miss many hits but serves as an initial filter to remove some semi-duplicate neighbourhoods. Depending on the number of sequences that remain, more sensitive alignment modes and higher max_target_seqs values are used to further reduce the Sieve search space. Alignment identity and coverage were not specified, as these reduce DIAMOND run time – instead, we relied on the relatively low sensitivity of the intermediate alignment modes specified to miss low quality (<60% identity) alignments. The final search for each dataset used the "more-sensitive" DIAMOND alignment mode, alignment criteria of at least 40% identity (the minimum recommended by DIAMOND for "more-sensitive" alignment) and 50% query coverage, and a "dynamic_max_target_seqs" which automatically set max_target_seqs to either the user-supplied max_target_seqs value (default 200) or the number of neighbourhoods being sieved, whichever was larger. This served to filter low quality alignments that might be detected by the more sensitive alignment mode and ensured that all hits to a given protein query were discovered. Where the number of retained neighbourhoods remained over 1000 after the initial Sieve filters, indicating a large number of diverse neighbourhoods, max_target_seqs was set to 500 instead of being dynamically defined – see **Supplementary Table 1**, Case Study 1 (Actinorhodin) commands.

S3: Comparing SyntenyQC and CAGEcleaner

SyntenyQC Collect was used to gather 42.5kb neighbourhoods for each of the cblaster hits in the case studies described by De Vrieze et al. (2025), both before and after CAGEcleaner processing – all cblaster data was taken from the supplementary data of De Vrieze et al. (2025). A key focus of our benchmarks was how many redundant neighbourhoods were removed by SyntenyQC Sieve, compared to CAGEcleaner. This

behaviour will be somewhat dependent on neighbourhood size for SyntenyQC Sieve – if a collection of neighbourhoods each exactly define a BGC (or BGC subsection) homolog, one might expect those neighbourhoods to be considered entirely redundant. Conversely, if those same neighbourhoods are extended beyond the BGC termini, then one would expect variation to arise outside of the co-selected BGC genes, and for the neighbourhoods to be considered less redundant. To reduce the likelihood of neighbourhoods in our benchmarks appearing overly redundant, which would benefit our benchmarks in favour of SyntenyQC (by making neighbourhoods identified by CAGEcleaner appear overly redundant), neighbourhoods for both case studies were generated using a neighbourhood span (42.5kb) that was twice the known BGC span of Actinorhodin (MIBIG entry BGC0000194).

The resulting four hit sets (case study 1, case study 1 post-CAGEcleaner, case study 2, case study 2 post-CAGEcleaner) were then filtered using SyntenyQC Sieve. The Collect binary files and all commands are given in **Supplementary Data 3** and **Supplementary Data 4** respectively.

Supplementary Figures

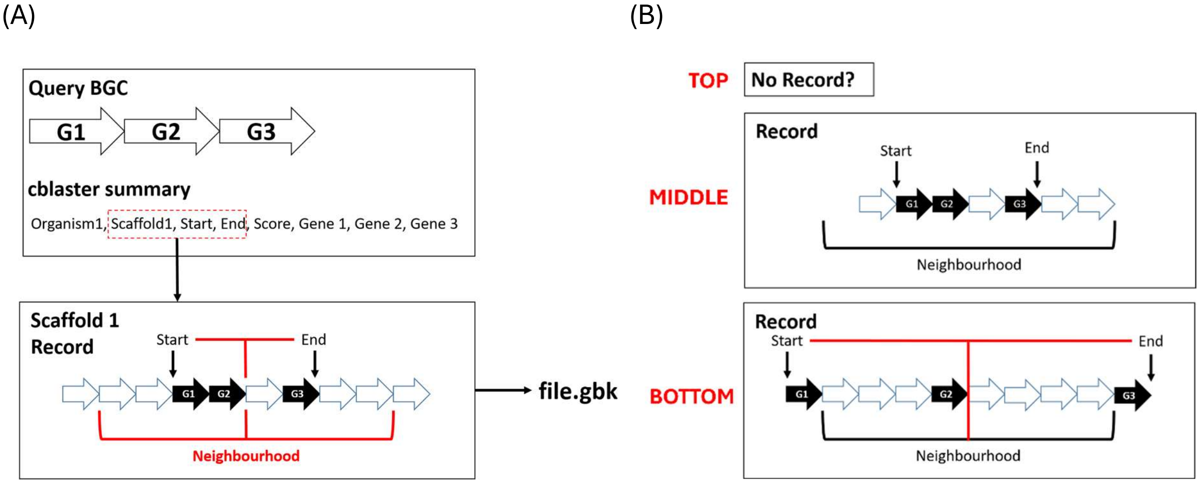

Supplementary Figure 1: The Collect subcommand. (A) The workflow. A neighbourhood is downloaded from NCBI using the accession number supplied in the cblaster result file. The loci of the first (Gene 1) and last (Gene 3) cluster gene homologs in this neighbourhood are used to define a neighbourhood of a user-specified size, with a mid-point that lies between the loci. This neighbourhood is then written to a local GenBank file. **(B) Rejected records.** The neighbourhood is rejected if (TOP) its accession is not recognised, (MIDDLE) making a neighbourhood of a user-specified size would

involve extending the neighbourhood beyond a contig edge – note this is optional, or (BOTTOM) if the hits identified by cblaster do not fall within a neighbourhood of user-specified size.

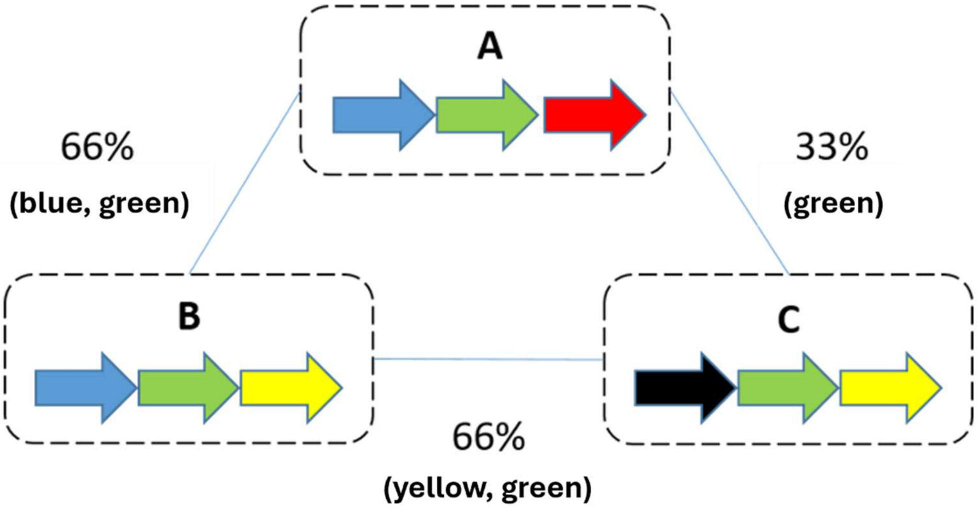

Supplementary Figure 2: Region similarity is non-transitive. Boxes indicate neighbourhoods, arrows indicate genes, colours indicate homolog groups. A/B and B/C are fairly similar in terms of homolog composition (66%), but A and C are much less similar (33%).

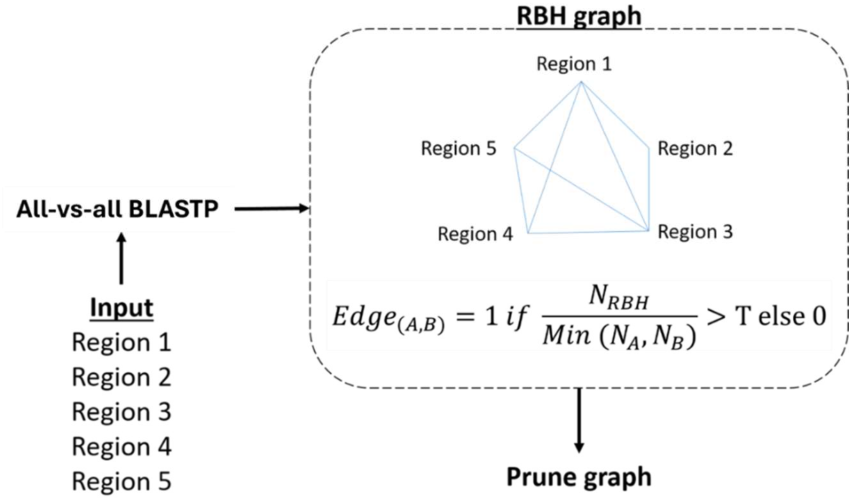

Supplementary Figure 3: The Sieve subcommand. A collection of input regions are subjected to an all-vs-all BLASTP to identify the number of reciprocal best hits (RBHs) between each pair of input regions. The neighbourhoods are then represented as a graph, where an edge is drawn between two nodes if their respective regions have a proportion of RBHs that exceed a user-defined threshold. N_{RBH} is the number of reciprocal best hits between two node regions (A and B), N_A and N_B are the number of proteins in regions A and B respectively. T is a threshold proportion that is set by the user (typically 0.5 to 0.7).

This graph is then pruned according to Supplementary Algorithm 1. Following pruning, the remaining neighbourhoods are returned to the user.

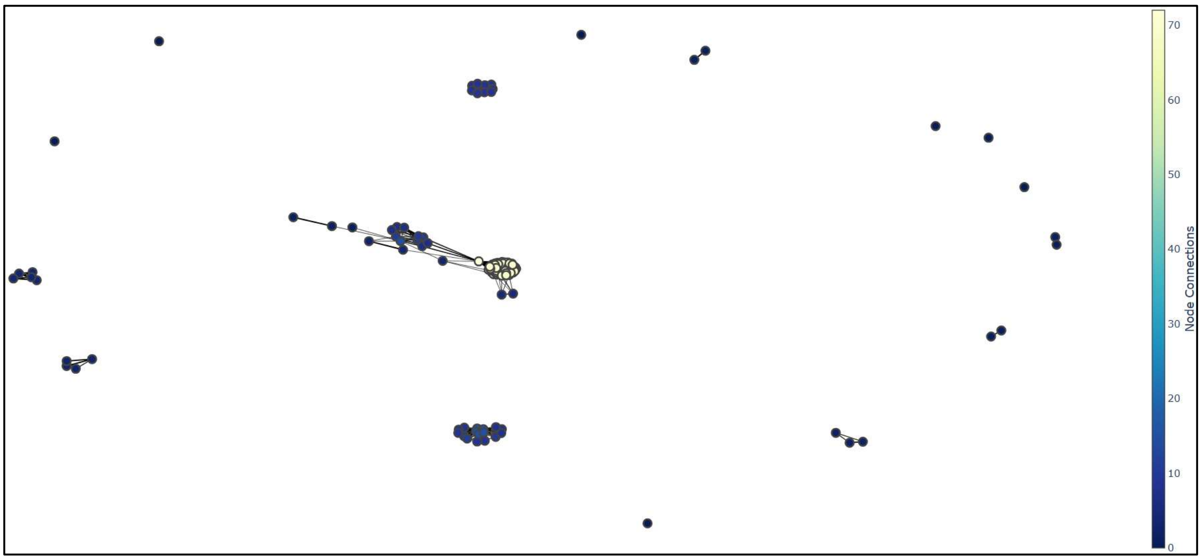

Supplementary Figure 4: The neighbourhood graph for actinorhodin MIBIG entry BGC000194. Shown is the neighbourhood graph described in Supplementary Figure 3, with neighbourhoods as nodes and edges indicating that two neighbourhoods have a similarity score that exceeds the user threshold. Prior to pruning via Supplementary Algorithm 1, the neighbourhood graph is written to a dynamic HTML file, with hover labels describing the details associated with each node and edge. Node colour indicates the degree of a given node, and edge thickness indicates the similarity score magnitude.

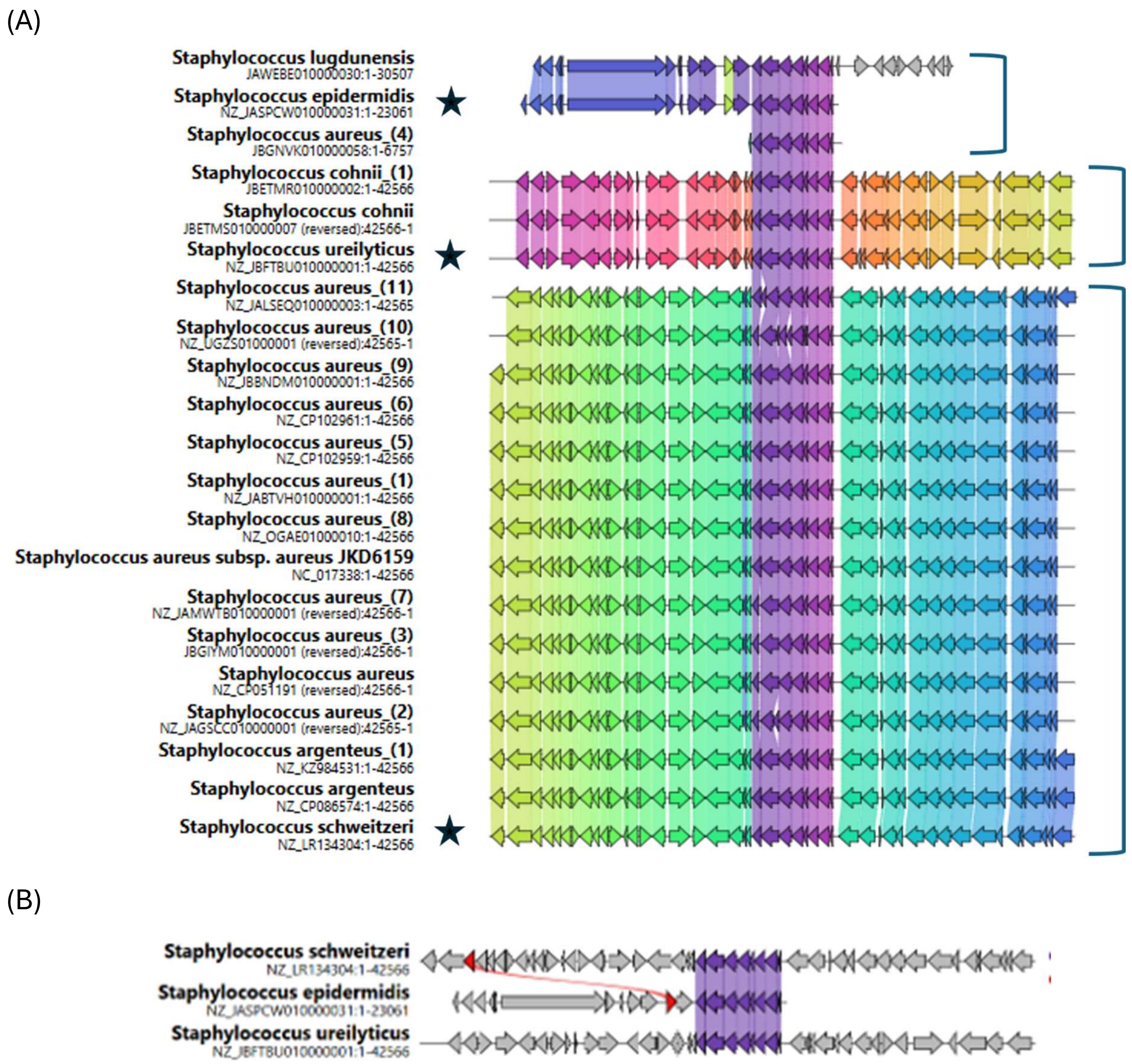

Supplementary Figure 5: Genomic variation does not guarantee neighbourhood variation. (A) CAGEcleaner. SyntenyQC Collect was used to gather 42.5kb neighbourhoods centred on the cblaster hits identified by De Vrieze et al. (2025), case 2, after processing with CAGEcleaner. Collect was run without strict span (i.e., neighbourhoods from records smaller than 42.5kb were accepted) to simplify comparisons with CAGEcleaner. Stars indicate neighbourhoods that remained after filtering with SyntenyQC Sieve in (B). Brackets indicate neighbourhood groups that were reduced to a single starred region following filtering with SyntenyQC Sieve, identified via visual inspection of the plots (note – "Staphylococcus aureus_(4)" could belong to any of the merged groups). Organism names (in bold) and NCBI accessions are automatically generated via SyntenyQC Collect. A small, putative BGC can be observed in purple. **(B) SyntenyQC Sieve**. The Neighbourhoods from (A) were processed with SyntenyQC Sieve to generate a less redundant synteny plot, where the putative BGC can be clearly defined (purple).

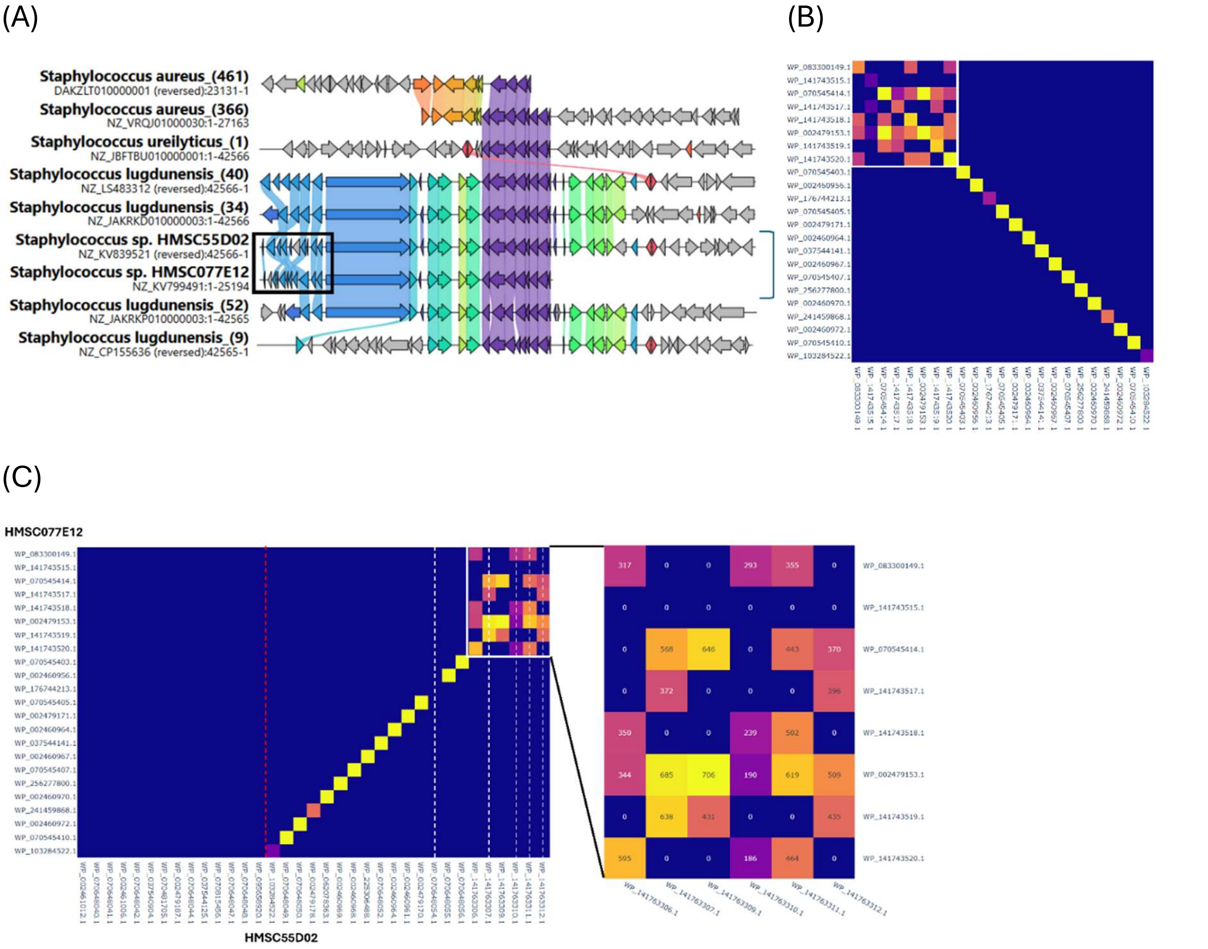

Supplementary Figure 6: SyntenyQC Sieve filtering can be less effective on neighbourhoods with high levels of internal redundancy. (A) Synteny plot showing neighbourhoods that survived filtering with SyntenyQC sieve. Neighbourhoods are taken from Case Study 2 (De Vrieze et al., 2025), and were filtered with SyntenyQC Sieve but not CAGEcleaner. *Staphylococcus sp. HMSC55D02* is an almost perfect superset of *Staphylococcus sp. HMSC077E12* (black bracket) and so should have been removed by Sieve. Black square shows a stretch of proteins that show some homology to other proteins in the same neighbourhood, which may have inhibited reciprocal best hit assignment during Sieve processing. **(B) Analysis of internal homology in *Staphylococcus sp. HMSC077E12.*** Colour intensity denotes bit score of local DIAMOND alignments, capped at 706 for visual clarity (the maximum bit score seen within the white box). White box corresponds to region in black box in (A). Protein IDs are given for each axis. Proteins without sequence information are excluded from DIAMOND analysis in Sieve. **(C) Analysis of local DIAMOND alignments between Staphylococcus sp. HMSC55D02 (X-axis) and Staphylococcus sp. HMSC077E12 (Y-axis).** Proteins with no reciprocal best hit are denoted by white dashed lines, unless they are upstream of the upstream terminus of Staphylococcus sp. HMSC077E12 (red dashed line). Region shown to right is the region encompassed by black and white boxes in (A) and (B) respectively. Numbers are bit scores for the alignment defined by DIAMOND between a given pair of proteins, capped at 706.

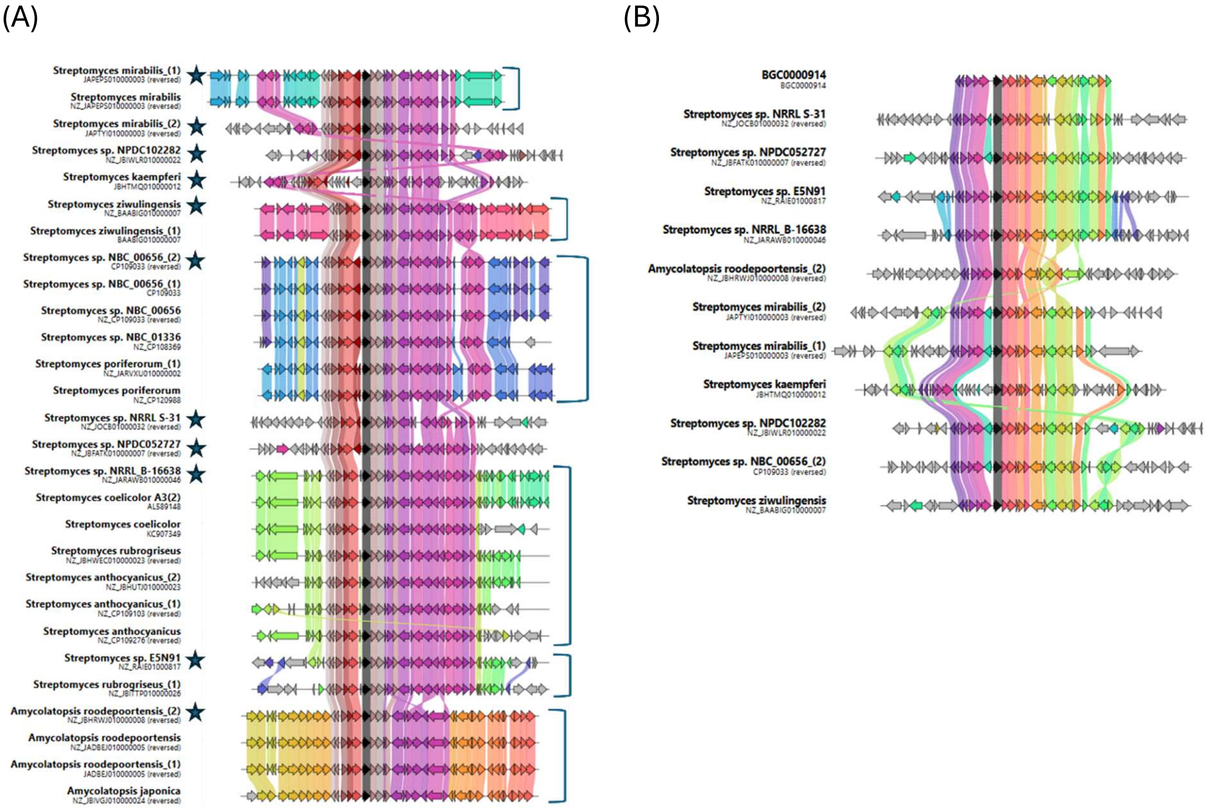

Supplementary Figure 7: Potential Information loss associated with the Sieve Algorithm. (A) Neighbourhoods identified via SyntenyQC Collect. Stars indicate neighbourhoods that remained after filtering with SyntenyQC Sieve in (B). Brackets indicate neighbourhood groups that were reduced to a single starred region following filtering with SyntenyQC Sieve, identified via visual inspection of the plots. Organism names (in bold) and NCBI accessions are automatically generated via SyntenyQC Collect. **(B) Neighbourhoods remaining after SyntenyQC Sieve.**

Supplementary Tables

Supplementary Table 1: Performance metrics for SyntenyQC. "Reference" indicates the relevant section for a given command. "Command" indicates SyntenyQC commands as used in the command line, omitting "—email" and full file paths for brevity – note, the binary files used by the Collect commands are given in Supplementary Data 3. "Neighbourhood count" indicates the number of neighbourhoods generated following a command. "Time" indicates time taken to run the command. "Disk Space" indicates the space requirements of a given command's output files, not including the temporary files generated by DIAMOND.

Reference	Command	Neighbourhood count	Time (hours:minutes:seconds)	Disk Space (MB)
Supplementary Methods S1	syntenyqc collect<br>-bp<br>...\BGC0000038_binary.csv<br>-ns 115774<br>-sp	28	00:11:35	7
	syntenyqc sieve<br>-gf<br>.../BGC0000038_binary/neighbourhood<br>-am fast<br>-mts 25	4	00:00:56	14.8
	syntenyqc sieve<br>-gf<br>.../BGC0000038_binary/neighbourhood/sieve_results/genbank<br>-am more-sensitive<br>-id 40<br>-qc 50<br>-dmts	1	00:00:16	9.7
	syntenyqc collect<br>-bp<br>...\BGC0000914_binary.csv<br>-ns 38684<br>-sp	28	00:05:10	2.7
	syntenyqc sieve<br>-gf<br>.../BGC0000914_binary/neighbourhood<br>-am fast<br>-mts 25	11	00:00:09	12.1

Supplementary Table 1 (Continued)

Reference	Command	Neighbourhood count	Time (hours:minutes:seconds)	Disk Space (MB)
Supplementary Methods S1	syntenyqc sieve<br>-gf<br>.../BGC0000914_binary/neighbourhood/sieve_results/genbank<br>-am more-sensitive<br>-id 40<br>-qc 50<br>-dmts	11	00:00:04	10.7
	syntenyqc collect<br>-bp<br>...\BGC0000194_binary.csv<br>-ns 42566<br>-sp	139	00:29:27	14
	syntenyqc sieve<br>-gf<br>.../BGC0000194_binary/neighbourhood<br>-am fast<br>-mts 25	78	00:01:19	27.6
	syntenyqc sieve<br>-gf<br>.../BGC0000194_binary/neighbourhood/sieve_results/genbank<br>-am more-sensitive<br>-id 40<br>-qc 50<br>-dmts	22	00:02:18	24.6
	syntenyqc collect<br>-bp<br>...\BGC0000315_binary.csv<br>-ns 165744<br>-sp	124	00:34:47	45.1
	syntenyqc sieve<br>-gf<br>.../BGC0000315_binary/neighbourhood<br>-am fast<br>-mts 25	62	00:02:52	62.2

Supplementary Table 1 (Continued)

Reference	Command	Neighbourhood count	Time (hours:minutes:seconds)	Disk Space (MB)
Supplementary Methods S1	syntenyqc sieve<br>-gf<br>.../BGC0000315_binary/neighbourhood/sieve_results/genbank<br>-am more-sensitive<br>-id 40<br>-qc 50<br>-dmts	13	00:05:43	47.8
	syntenyqc collect<br>-bp<br>...\BGC0001063_binary.csv<br>-ns 63282<br>-sp	325	01:05:42	47.1
	syntenyqc sieve<br>-gf<br>.../BGC0001063_binary/neighbourhood<br>-am fast<br>-mts 25	128	00:04:11	66.8
	syntenyqc sieve<br>-gf<br>.../BGC0001063_binary/neighbourhood/sieve_results/genbank<br>-am more-sensitive<br>-id 40<br>-qc 50<br>-dmts	36	00:05:56	72.2
	syntenyqc collect<br>-bp<br>...\BGC0000551_binary.csv<br>-ns 13456<br>-sp	541	01:02:11	17.9
	syntenyqc sieve<br>-gf<br>.../BGC0000551_binary/neighbourhood<br>-am fast<br>-mts 25	184	00:01:40	28.5

Supplementary Table 1 (Continued)

Reference	Command	Neighbourhood count	Time (hours:minutes:seconds)	Disk Space (MB)
Supplementary Methods S1	syntenyqc sieve<br>-gf<br>.../BGC0000551_binary/neighbourhood/sieve_results/genbank<br>-am more-sensitive<br>-id 40<br>-qc 50<br>-dmts	28	00:02:49	34.2
	syntenyqc collect<br>-bp<br>...\BGC0000325_binary.csv<br>-ns 45252<br>-sp	542	00:53:56	55.4
	syntenyqc sieve<br>-gf<br>.../BGC0000325_binary/neighbourhood<br>-am fast<br>-mts 25	218	00:06:56	73.7
	syntenyqc sieve<br>-gf<br>.../BGC0000325_binary/neighbourhood/sieve_results/genbank<br>-am more-sensitive<br>-id 40<br>-qc 50<br>-dmts	45	00:16:13	110
	syntenyqc collect<br>-bp<br>...\BGC0000324_binary.csv<br>-ns 50242<br>-sp	612	01:09:51	70.1
	syntenyqc sieve<br>-gf<br>.../BGC0000324_binary/neighbourhood<br>-am fast<br>-mts 25	240	00:07:40	90

Supplementary Table 1 (Continued)

Reference	Command	Neighbourhood count	Time (hours:minutes:seconds)	Disk Space (MB)
Supplementary Methods S1	syntenyqc sieve<br>-gf<br>.../BGC0000324_binary/neighbourhood/sieve_results/genbank<br>-am more-sensitive<br>-id 40<br>-qc 50<br>-dmts	54	00:15:01	139
	syntenyqc collect<br>-bp<br>...\BGC0000940_binary.csv<br>-ns 14656<br>-sp	738	01:17:45	26.4
	syntenyqc sieve<br>-gf<br>.../BGC0000940_binary/neighbourhood<br>-am fast<br>-mts 25	314	00:05:32	39.9
	syntenyqc sieve<br>-gf<br>.../BGC0000940_binary/neighbourhood/sieve_results/genbank<br>-am more-sensitive<br>-id 40<br>-qc 50<br>-dmts	3	00:14:52	82.6
	syntenyqc collect<br>-bp<br>...\BGC0000910_binary.csv<br>-ns 2840<br>-sp	747	01:32:11	8.7
	syntenyqc sieve<br>-gf<br>.../BGC0000910_binary/neighbourhood<br>-am fast<br>-mts 25	316	00:01:50	20.3

Supplementary Table 1 (Continued)

Reference	Command	Neighbourhood count	Time (hours:minutes:seconds)	Disk Space (MB)
Supplementary Methods S1	syntenyqc sieve<br>-gf<br>.../BGC0000910_binary/neighbourhood/sieve_results/genbank<br>-am more-sensitive<br>-id 40<br>-qc 50<br>-dmts	4	00:05:06	44.9
	syntenyqc collect<br>-bp<br>...\BGC0002127_binary.csv<br>-ns 6028<br>-sp	769	01:39:56	13.3
	syntenyqc sieve<br>-gf<br>.../BGC0002127_binary/neighbourhood<br>-am fast<br>-mts 25	445	00:02:37	26.5
	syntenyqc sieve<br>-gf<br>.../BGC0002127_binary/neighbourhood/sieve_results/genbank<br>-am more-sensitive<br>-id 40<br>-qc 50<br>-dmts	3	00:15:14	82.3
	syntenyqc collect<br>-bp<br>...\BGC0000663_binary.csv<br>-ns 27514<br>-sp	656	01:48:08	46.1
	syntenyqc sieve<br>-gf<br>.../BGC0000663_binary/neighbourhood<br>-am fast<br>-mts 25	392	00:07:56	67.8

Supplementary Table 1 (Continued)

Reference	Command	Neighbourhood count	Time (hours:minutes:seconds)	Disk Space (MB)
Supplementary Methods S1	syntenyqc sieve<br>-gf<br>.../BGC0000663_binary/neighbourhood/sieve_results/genbank<br>-am more-sensitive<br>-id 40<br>-qc 50<br>-dmts	1	00:45:38	160.9
	syntenyqc collect<br>-bp<br>...\BGC0002128_binary.csv<br>-ns 17344<br>-sp	798	01:15:05	37.9
	syntenyqc sieve<br>-gf<br>.../BGC0002128_binary/neighbourhood<br>-am fast<br>-mts 25	365	00:06:53	54.5
	syntenyqc sieve<br>-gf<br>.../BGC0002128_binary/neighbourhood/sieve_results/genbank<br>-am more-sensitive<br>-id 40<br>-qc 50<br>-dmts	2	00:24:02	124
	syntenyqc collect<br>-bp<br>...\BGC0000660_binary.csv<br>-ns 4936<br>-sp	927	01:32:19	14.7
	syntenyqc sieve<br>-gf<br>.../BGC0000660_binary/neighbourhood<br>-am fast<br>-mts 25	341	00:03:23	26.8

Supplementary Table 1 (Continued)

Reference	Command	Neighbourhood count	Time (hours:minutes:seconds)	Disk Space (MB)
Supplementary Methods S1	syntenyqc sieve<br>-gf<br>.../BGC0000660_binary/neighbourhood/sieve_results/genbank<br>-am more-sensitive<br>-id 40<br>-qc 50<br>-dmts	1	00:07:50	53.5
	syntenyqc collect<br>-bp<br>...\BGC0000849_binary.csv<br>-ns 3420<br>-sp	954	05:20:24	10.5
	syntenyqc sieve<br>-gf<br>.../BGC0000849_binary/neighbourhood<br>-am fast<br>-mts 25	334	00:02:26	20.6
	syntenyqc sieve<br>-gf<br>.../BGC0000849_binary/neighbourhood/sieve_results/genbank<br>-am more-sensitive<br>-id 40<br>-qc 50<br>-dmts	4	00:04:50	42.6
	syntenyqc collect<br>-bp<br>...\BGC0001181_binary.csv<br>-ns 4362<br>-sp	988	06:00:24	12.2
	syntenyqc sieve<br>-gf<br>.../BGC0001181_binary/neighbourhood<br>-am fast<br>-mts 25	267	00:03:11	23.2

Supplementary Table 1 (Continued)

Reference	Command	Neighbourhood count	Time (hours:minutes:seconds)	Disk Space (MB)
Supplementary Methods S1	syntenyqc sieve<br>-gf<br>.../BGC0001181_binary/neighbourhood/sieve_results/genbank<br>-am more-sensitive<br>-id 40<br>-qc 50<br>-dmts	9	00:03:20	33.1
	syntenyqc collect<br>-bp<br>...\BGC0000595_binary.csv<br>-ns 43542<br>-sp	693	04:46:44	68.6
	syntenyqc sieve<br>-gf<br>.../BGC0000595_binary/neighbourhood<br>-am fast<br>-mts 25	314	00:10:32	91.3
	syntenyqc sieve<br>-gf<br>.../BGC0000595_binary/neighbourhood/sieve_results/genbank<br>-am more-sensitive<br>-id 40<br>-qc 50<br>-dmts	29	00:29:05	172.2
Supplementary Methods S3	syntenyqc collect<br>-bp<br>.../CageCleaner_act.csv<br>-ns 42566	8933	12:29:20	800
	syntenyqc sieve<br>-gf<br>.../CageCleaner_act/neighbourhood<br>-am fast<br>-mts 25	3006	03:58:32	1400
	syntenyqc sieve<br>-gf<br>.../CageCleaner_act/neighbourhood/sieve_results/genbank	1508	02:28:33	2000

Supplementary Table 1 (Continued)

Reference	Command	Neighbourhood count	Time (hours:minutes:seconds)	Disk Space (MB)
Supplementary Methods S3	syntenyqc sieve -gf .../CageCleaner_act/neighbourhood/sieve_results/genbank/sieve_results/genbank -am more-sensitive -id 40 -qc 50 -mts 500	1434	01:29:10	1700
	syntenyqc collect -bp .../CageCleaner_act_filtered.csv -ns 42566	4846	07:47:27	400
	syntenyqc sieve -gf .../CageCleaner_act_filtered/neighbourhood -am fast -mts 25	1849	01:16:18	900
	syntenyqc sieve -gf .../CageCleaner_act_filtered/neighbourhood/sieve_results/genbank	1444	01:13:05	1600
	syntenyqc sieve -gf .../CageCleaner_act_filtered/neighbourhood/sieve_results/genbank/sieve_results/genbank -am more-sensitive -id 40 -qc 50 -mts 500	1388	01:18:57	1600
	syntenyqc collect -bp .../CageCleaner_staph.csv -ns 42566	1145	01:07:32	116.2

Supplementary Table 1 (Continued)

Reference	Command	Neighbourhood count	Time (hours:minutes:seconds)	Disk Space (MB)
Supplementary Methods S3	syntenyqc sieve -gf .../CageCleaner_staph/neighbourhood -am fast -mts 25	759	00:24:41	163.5
	syntenyqc sieve -gf .../CageCleaner_staph/neighbourhood/sieve_results/genbank -am more-sensitive -id 40 -qc 50 -dmts	9	03:44:55	486
	syntenyqc collect -bp .../CageCleaner_staph_filtered.csv -ns 42566	21	00:01:37	2.1
	syntenyqc sieve -gf .../CageCleaner_staph_filtered/neighbourhood -am more-sensitive -id 40 -qc 50 -dmts	3	00:00:14	10.5

Supplementary Algorithms

Algorithm 1

Data: RBH graph

Result: Nodes from pruned RBH graph

Procedure:

    while max(*node degrees* in *RBH graph*) > 0:

        delete nodes = []

        for *node* in *RBH graph*:

            if *node degree* = **max**(*node degrees* in *RBH graph*):

                delete nodes + node

        delete node = random node from *delete nodes*

        RBH graph = RBH graph - delete node

    return *nodes* in *RBH graph*

Supplementary Bibliography

De Vrieze, L., Biltjes, M., Lukashevich, S., Tsurumi, K., & Masschelein, J. (2025). CAGEcleaner: reducing genomic redundancy in gene cluster mining. *Bioinformatics*, *41*(7), btaf373. https://doi.org/10.1093/bioinformatics/btaf373

Gilchrist, C. L. M., Booth, T. J., Van Wersch, B., Van Grieken, L., Medema, M. H., & Chooi, Y. H. (2021). cblaster: a remote search tool for rapid identification and visualization of homologous gene clusters. *Bioinformatics Advances*, *1*(1). https://doi.org/10.1093/BIOADV/VBAB016

Gilchrist, C. L. M., & Chooi, Y. H. (2021). clinker & clustermap.js: automatic generation of gene cluster comparison figures. *Bioinformatics*, *37*(16), 2473–2475. https://doi.org/10.1093/BIOINFORMATICS/BTAB007

Zdouc, M. M., Blin, K., Louwen, N. L., Navarro, J., Loureiro, C., Bader, C. D., Bailey, C. B., Barra, L., Booth, T. J., Bozhueyuek, K. A., Cediel-Becerra, J. D., Charlop-Powers, Z., Chevrette, M. G., Chooi, Y. H., D'Agostino, P. M., de Rond, T., Pup, E. D., Duncan, K. R., Gu, W., ... Dillen, J. (2025). MIBiG 4.0: advancing biosynthetic gene cluster curation through global collaboration. *Nucleic Acids Research*, *53*(D1), 678–690. https://doi.org/10.1093/nar/gkae1115
